# Assessment of intraocular foreign body using high resolution 3D ultrasound imaging

**DOI:** 10.1038/s41598-024-62362-y

**Published:** 2024-05-26

**Authors:** Ahmed Tahseen Minhaz, Faruk H. Orge, David L. Wilson, Mahdi Bayat

**Affiliations:** 1 Department of Biomedical Engineering, Case Western Reserve University, Cleveland, OH USA; 2 Department of Ophthalmology and Visual Sciences, Case Western Reserve University, Cleveland, OH USA; 3 Department of Electrical, Computer and Systems Engineering, Case Western Reserve University, Cleveland, OH USA; 4 Department of Radiology, Case Western Reserve University, Cleveland, OH USA; 5 Department of Pediatric Ophthalmology and Adult Strabismus, University Hospitals, Cleveland, OH USA

**Keywords:** Eye diseases, Trauma, Biomedical engineering

## Abstract

Ocular trauma often involves intraocular foreign bodies (IOFBs) that pose challenges in accurate diagnosis due to their size, shape, and material composition. In this study, we proposed a novel whole-eye 3D ophthalmic ultrasound B-scan (3D-UBS) system for automating image acquisition and improved 3D visualization, thereby improving sensitivity for detecting IOFBs. 3D-UBS utilizes 14 MHz Clarius L20 probe, a motorized translation stage, and a surgical microscope for precise placement and movement. The system’s 3D point spread function (PSF) is 0.377 × 0.550 × 0.894 mm^3^ characterized by the full-width at half-maximum intensity values in the axial, lateral and elevation directions. Digital phantom and ex vivo ocular models were prepared using four types of IOFBs (i.e., plastic, wood, metal, and glass). Ex vivo models were imaged with both 3D-UBS and clinical computed tomography (CT). Image preprocessing was performed on 3D-UBS images to remove uneven illumination and speckle noise. Multiplanar reformatting in 3D-UBS provides optimal plane selection after acquisition, reducing the need for a trained ultrasonographer. 3D-UBS outperforms CT in contrast for wood and plastic, with mean contrast improvement of 2.43 and 1.84 times, respectively. 3D-UBS was able to identify wood and plastic IOFBs larger than 250 µm and 300 in diameter, respectively. CT, with its wider PSF, was only able to detect wood and plastic IOFBs larger than 600 and 550 µm, respectively. Although contrast was higher in CT for metal and glass IOFBs, 3D-UBS provided sufficient contrast to identify those. 3D-UBS provides an easy-to-use, non-expert imaging approach for identifying small IOFBs of different materials and related ocular injuries at the point of care.

## Introduction

Ocular injuries affect individuals across various contexts, including occupational accidents, active duty, sports, and everyday activities. According to The National Institute for Occupational Safety and Health, in 2021, ocular injuries accounted for 5.1% of the overall work-related injury, totaling over 97,500 cases. Among the ocular injuries, foreign body was responsible for over 29,700 cases (30.5%)^1^. Foreign bodies are also commonly seen in combat ocular trauma and associated with severe injuries^2^. Traumatic incidents such as explosions, accidents, or high-velocity projectiles can lead to ocular injuries that range from direct tissue damage to the propulsion of intraocular foreign bodies (IOFBs). In both scenarios, the vulnerability of delicate ocular structures is evident, with retinal detachment emerging as a particularly critical outcome, potentially resulting in irreversible vision loss. The presence of blood, severe edema, or disorganized opaque tissue often complicates the use of conventional imaging methods like direct ophthalmoscopy and optical computed tomography (OCT). Even commonly employed, thin-slice computed tomography (CT) can fall short in identifying small-scale injuries, such as retinal detachment, or detecting non-metallic IOFBs^3,4^, which necessitate swift diagnosis and intervention. Moreover, precise localization of IOFBs is pivotal for successful surgical removal, yet the clarity of CT imaging near the scleral wall can be inadequate^4^.

Conventionally, ultrasound has been used alongside CT as a reliable adjunct, especially for detecting subtle intraocular foreign bodies (IOFBs)^3–7^. This is evident in data from Walter Reed Army Medical Center, where 121 (28%) out of 432 ocular injury cases between 2003 and 2006 underwent B-scan ultrasound^8^, underscoring the critical role of ultrasound in diagnosing and managing ocular trauma. However, conventional ultrasound techniques demand direct contact and skilled probe maneuvering, which may not be ideal for cases involving potentially perforated globes^9^. Furthermore, the higher incidence of secondary blast injuries and the growing likelihood of perforating and penetrating IOFBs underscore the necessity for a streamlined solution that will reduce operator dependency and minimize the risk of secondary damage or infection^10,11^.

To address this critical gap in ocular diagnostics, we propose the development of a novel 3D ophthalmic Ultrasound B-Scan (3D-UBS) system that will:

- Automate 3D acquisition to eliminate the need for highly specialized operators and risky probe maneuvers in conventional 2D ultrasound, especially on injured eyes.
- Provide intuitive, interactive 3D visualization of ocular structures, IOFBs etc. including en face and oblique views using multiplanar reformatting that conventional 2D ultrasound cannot achieve.
- Provide improved visualization of small, low-density material IOFBs not visible with clinical CT.

## Materials and methods

### 3D ultrasound B-scan (3D-UBS) system design

**Figure 1:**
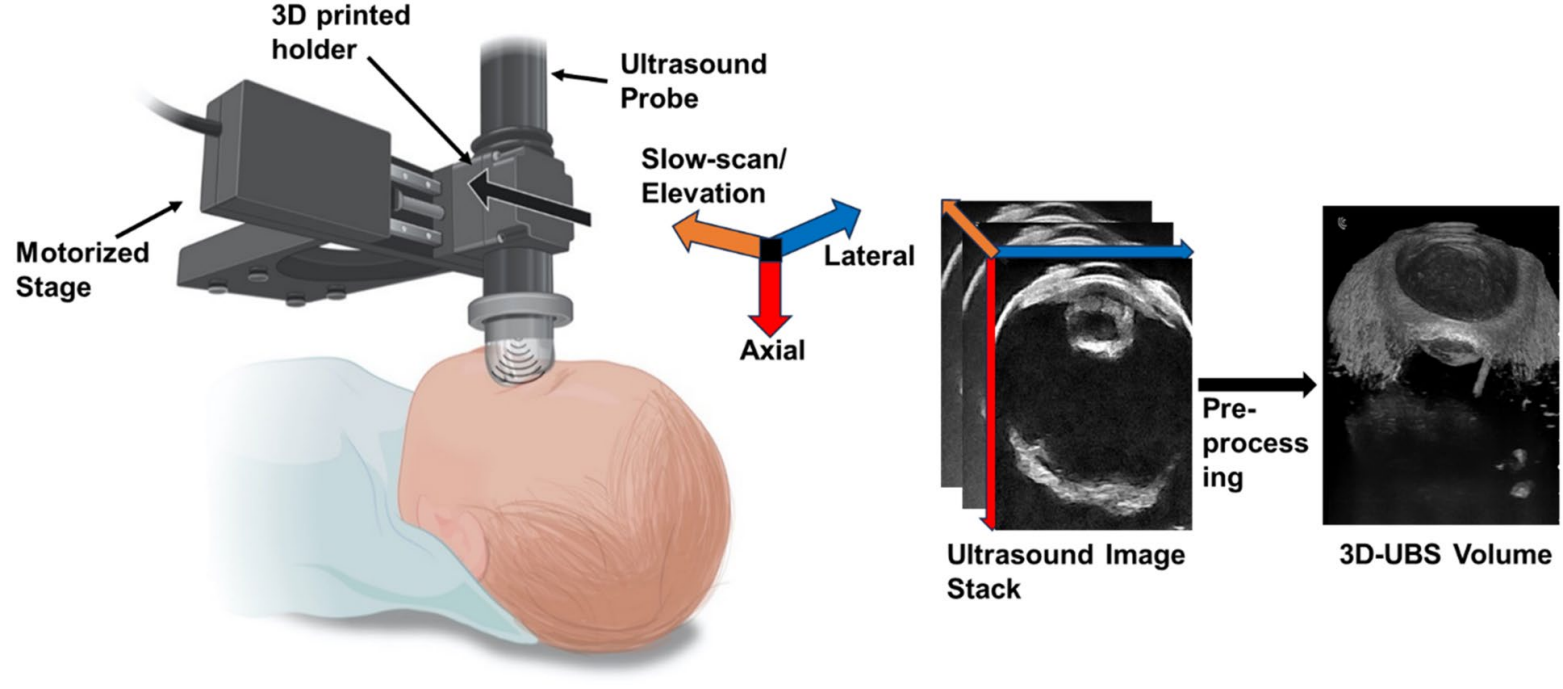
3D ophthalmic Ultrasound B-scan (3D-UBS) system. Ultrasound probe is translated across the eye using a motorized stage and surgical microscope (not shown) to acquire a 2D image stack along the slow scan direction. Images are pre-processed and converted into a 3D-UBS volume.

We developed our 3D ultrasound system using the Clarius L20 HD3 probe (Center frequency: 14 MHz) in conjunction with a motorized translation stage (MTS50-Z8, Thorlabs Inc.) and a surgical microscope (Fig. 1). The probe was attached to a custom 3D printed holder to the translation stage, which was coupled to the microscope for precise movement. The probe was acoustically coupled to the eye using a water-filled pliable chamber which provided a safe scanning distance between the eye and the probe surface. The motor moves the probe across the eye at a constant speed, enabling image acquisition in the slow scan direction (slow-axis or elevation plane). Images can be acquired by a non-expert, as manual maneuvering of the probe to find the best plane for image acquisition is not necessary. Motor speed is determined by the length of the eye in the slow scan direction and the maximum acquisition time available by the Clarius system. The motor movement control software provided the output signal to control the footswitch, which was used to synchronize the image scan. Each image acquired is in the 2D axial-lateral plane. The system was calibrated to acquire images for 30 s, the maximum allowed by the Clarius imaging software. The probe is capable of providing both radio-frequency (RF) data and B-mode images. B-mode images were exported as MPEG-4 movie files of resolution 3840 × 2160 × 720 (no. of frames). Each frame from the movie was processed to crop out the dark border, containing no information. Using the stack of image frames, we can create the entire 3D volume of the eye. The dimension of each volume was determined by the frame axial depth chosen by the user and the length of the eye in the slow scan direction. A 3D acquisition typically consists of 19 µm × 19 µm pixel frames with nominally 35 µm spacing (slow axis), giving 720, 1587 × 1329 pixel image frames across a 25 mm eye.

### Point spread function (PSF) assessment

**Figure 2:**
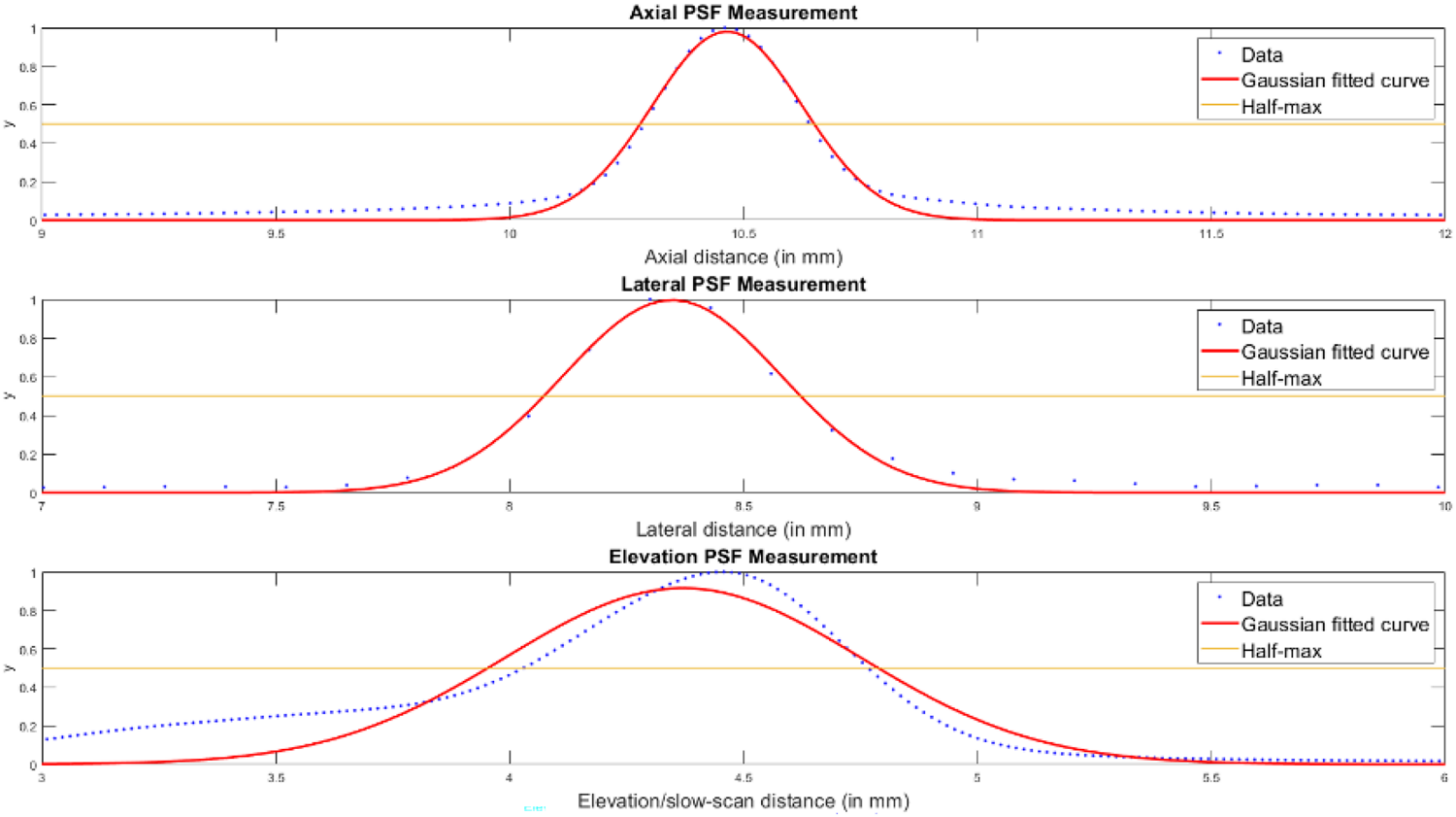
PSF characterization of the 3D-UBS system with Clarius L20 HD3 probe. The PSF of the 3D ultrasound system was assessed using a 40 µm wire phantom in water bath as a point scatterer. Echo data were acquired along the axial, lateral, and elevation directions, averaged, and normalized. Full Width Half Max (FWHM) PSFs in each direction were determined by fitting a Gaussian curve to the data, measuring the physical distance at which the ultrasound echo reached half of its peak value.

Point spread function (PSF) is the response of an imaging system to a point input. PSF describes the extent of blurring introduced by the imaging system during acquisition or reconstruction and is a measure of spatial resolution^10^. For two-dimensional (2D) ultrasound, PSF is a 2D function, described in the axial and lateral direction. 3D-UBS has a 3D-PSF, with measurements along three directions: axial, lateral, and elevation. Wire phantoms have been used previously to determine PSF of ultrasound imaging systems^11,12^. Wire phantom was created by placing two 40 µm wires perpendicular to each other, with two mm separation between them. The phantom was submerged in water and scanned using the Clarius probe. Imaging depth and focal depth were set to 24.9 and 10 mm respectively. Imaging width was 24.93 mm. From each frame of the RF data, we calculated the number of lines (L = 192), and samples per line (Ns = 976). Clarius probe has a center transmit frequency of 14 MHz and a sampling frequency of 30 MHz. From the RF data of the wire phantom, we extracted the axial,lateral and elevation line intensity profiles of the point object (wire) along the brightest point in the image. Gaussian models were fit to all theintensity profiles. Axial,lateral and elevation resolutions were measured by measuring the distances at which the peak intensities were halved. This is also known as the full width at half maximum (FWHM) of the PSF. From our experiments, mean axial, lateral, and elevation PSFs were measured at 377, 550, and 894 µm respectively, giving our system sub-millimeter resolution in all three axes (Fig. 2).

### Digital phantom preparation

To compare ultrasound’s performance against CT, we created digital phantoms consisting of small circular IOFBs (e.g., plastic, wood, glass, and metal) of increasing diameters, placed in vitreous humor. The CT digital phantom, ${I}_{dp-CT}$, was created using material-specific linear attenuation coefficients, as obtained from Hounsfield units (HU). Hounsfield units for associated materials are shown in Table 1. Values within IOFB disk and vitreous humor in ${I}_{dp-CT}$ were randomly sampled from normal distributions with material-specific HU mean and standard deviation (SD). To create an acoustic digital phantom ${I}_{dp-US}$, first B-mode ultrasound images of IOFBs of different materials were acquired and deconvolved using Lucy-Richardson with empirical $PS{F}_{3D-UBS}$. Table 1 also shows an improved estimation of gray-scale intensity or tissue response of the IOFB materials and vitreous humor without the effect of PSF. An acoustic digital phantom of IOFB in vitreous ${I}_{dp-US}$, was created by randomly sampling grayscale values from IOFB and vitreous-specific normal distribution.

**Table 1:** Material properties of vitreous and different IOFBs for digital phantom creation.

Type	Hounsfield Unit, experimental (mean ± SD)	Grayscale intensity in ultrasound, experimental (mean ± SD)
Vitreous humor	15 ± 22	78 ± 11
Plastic (polylactic acid, PLA)	− 20 ± 27	212 ± 39
Wood (porous)	40 ± 43	227 ± 41
Glass (metallic coating)	2340 ± 570	235 ± 13
Metal (stainless steel)	1817 ± 459	244 ± 7

Simulated CT and ultrasound images were created using the following formulas.

1 $${I}_{CT}={I}_{dp-CT}*PS{F}_{CT}+{N}_{CT}$$

2 $${I}_{US}={I}_{dp-US}*PS{F}_{3D-UBS}*+{N}_{US}$$

where ${N}_{CT}$ and ${N}_{US}$ are additive noise in the CT and ultrasound systems respectively. $PS{F}_{3D-UBS}$ and, and $PS{F}_{CT}$ are experimental PSFs of 3D-UBS and clinical CT systems respectively. FWHM $PS{F}_{CT}$ was estimated as 1.31 × 1.31 mm^2^ for the same clinical CT scanner (Siemens SOMATOM Definition Flash, Siemens Healthcare, Erlangen, Germany) used for ex vivo imaging^13^.

### Ex vivo ocular model preparation

Ex vivo porcine eyes were used for our experiments. Fresh ex vivo eyes with optic nerve attached were sourced from a commercial abattoir (Animal Technologies Inc., Tyler, Texas, USA). Eyes were transported in saline, to reduce any additional damage or injury. For IOFB models, IOFBs of different sizes and compositions (wood, metal, plastic, glass) were inserted into the eyes through small incisions made in the sclera. In cases where the IOFBs were completely placed inside the globes, the incisions were closed via suturing/glue. IOFB insertion and suturing were performed under a surgical microscope by an ophthalmology specialist with 18 years of experience in pediatric ophthalmology. In total, 11 porcine eyes were prepared (5× wood, 3× plastic, 2  glass and 1× metal IOFB). Only one IOFB was inserted in each eye for wood, plastic, and glass cases. For metal, two metal IOFBs were inserted in the eye model. The wooden IOFBs ranged from 6 to 10 mm in length and 1–3 mm in diameter. Plastic IOFB material was poly-lactic acid (PLA), commonly used as a 3D-printing filament. Plastic IOFB size ranged from 6 to 8 mm in length and 3 mm in diameter. Glass IOFBs were crushed mirrored glass chips with dimensions ranging between 2 and 5 mm. After inserting the IOFBs, eyes were embedded in a gelatin phantom. For phantom preparation, we adjusted the phantom recipe proposed by Nabavizadeh et. al according to our needs^14^. First, a 12% (w/v) solution of gelatin (Sigma-Aldrich, St. Louis, MO, USA) and water (60 °C) was prepared. The solution was cooled down to room temperature. As it started to solidify, the ex vivo eye was embedded in the mixture and additional solution was added to cover the entire eye. The eye-gelatin phantom was then refrigerated until solidification. Embedding in gelatin provided the ex vivo models necessary structural support and restricted movement of the samples between imaging experiments.

### Imaging experiments and dataset

We scanned 11 cadaver eyes containing IOFBs using both 3D-UBS and CT. Ultrasound imaging experiment was performed first using our 3D-UBS system, right after the phantoms were prepared to maintain tissue freshness. CT experiment was performed at University Hospitals Seidman Cancer Center, Cleveland, Ohio, USA, within 6 h of 3D-UBS imaging. The clinical CT system was Siemens SOMATOM Definition Flash (Siemens Healthcare, Erlangen, Germany). Head CT was performed with a tube voltage of 120 kVp, tube current of 100 mA, exposure of 125 mAs, and slice thickness of 0.75 mm. Each volume consisted of 126 slices of 512 × 512 images. CT volumes were of size ~ 96 mm × 96 mm × 50 mm, with a voxel size of 0.1875 × 0.1875 × 0.4 mm (slice interval).

### Image processing

Image processing was performed to remove any unwanted noise or artifacts, and to improve visualization. The image acquisition software creates a movie containing B-mode images as frames. Each frame contained padded empty space and highly reflective gelatin-probe boundary, that were removed via center cropping. Then we performed top-hat filtering with a large structural element on image frames to remove uneven illumination. Finally, median filtering was performed to reduce ultrasound speckle. Preprocessing and volume reconstruction were performed in MATLAB 2022b (MathWorks, Natwick, USA).

### Performance metrics

We demonstrated two commonly used metrics in image quality and object detectability analysis, namely contrast-to-noise ratio and Rose signal-to-noise ratio.

*Contrast to noise ratio (CNR)*: The contrast to noise ratio (CNR)^10,15^ is a size-independent measure of object or region-of-interest contrast in the presence of noise. CNR is defined as,

3 $$CNR= \frac{|{\mu }_{obj}-{\mu }_{bg}|}{{\sigma }_{bg}}$$

where ${\mu }_{obj}$ and ${\mu }_{bg}$ are mean, ${\sigma }_{bg}$ is the standard deviation of the object and background region in the image respectively.

*Rose signal-to-noise ratio* (SNR_Rose_): The Rose signal-to-noise ratio^16–18^ (SNR_Rose_) is a measure of object detectability with respect to the background noise, and takes into account the size of the object. SNR_Rose_ is defined as,

4 $${SNR}_{Rose}= \frac{|{\mu }_{obj}-{\mu }_{bg}|}{{\sigma }_{bg}} \times \sqrt{\frac{{A}_{obj}}{{A}_{pixel}}}$$

where ${\mu }_{obj}$ and ${\mu }_{bg}$ are mean values of the object and background region respectively, ${\sigma }_{bg}$ is the standard deviation of the noisy background region, ${A}_{obj}$ is the area of the object, and ${A}_{pixel}$ is the area of a pixel in the image. Rose criterion states that for an object to be detectable in most cases, ${SNR}_{Rose}$ has to be higher than five^10^.

## Results

### Effects of image preprocessing on visualization

**Figure 3:**
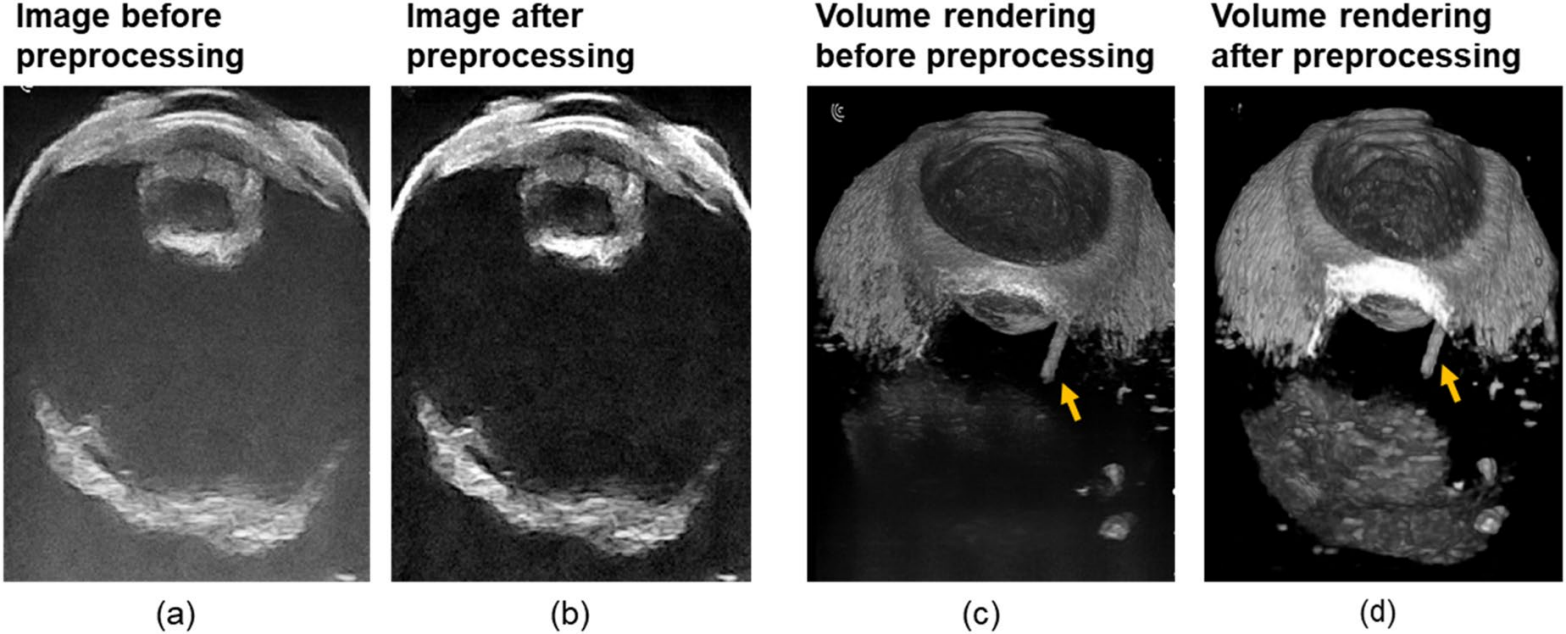
Image preprocessing for the 3D-UBS system. (**a**) and (**b**) shows images of a cadaver pig eye before and after preprocessing. Top-hat filtering was performed using a disk to reduce uneven illumination and median filtering was performed to reduce speckle noise. (**c**) and (**d**) show the effect of preprocessing in volume rendering. 3D visualization of the posterior region of the eye improved. The yellow arrow represents the wooden IOFB in the eye.

We imaged IOFBs of different materials (e.g., plastic, wood, glass, and metal) in ex vivo porcine eyes with both 3D-UBS and clinical CT systems. Cropping, top-hat filtering and median filtering were performed on images. Figure 3 shows the effect of top-hat filtering and median filtering in single image and volume rendering. After preprocessing, the uneven illumination and noise in the sample image were minimized, improving contrast. Volume rendering of an entire eye embedded with a wooden IOFB shows preprocessing improved visualization within the same window and level.

### Advantages of 3D-UBS over 2D ultrasound

**Figure 4:**
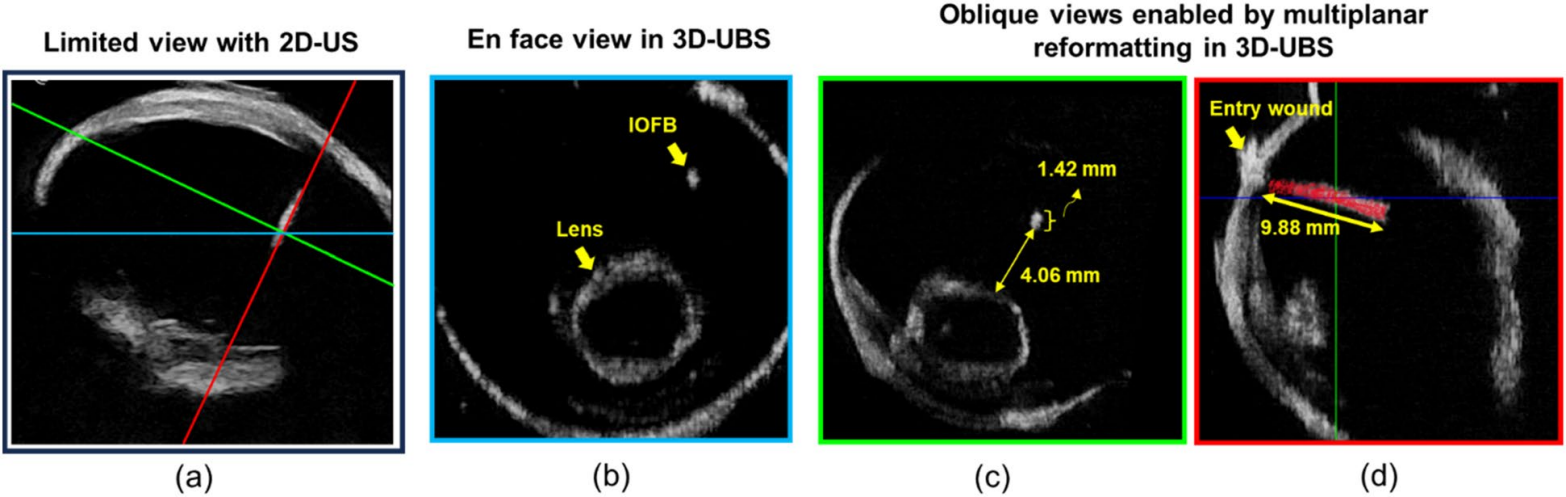
Visualization of a wooden IOFB in 3D-UBS. 2D ultrasound image (**a**) of the IOFB is limited in information. 3D-UBS allows en face view (blue-**b**) of the eye, not possible with 2D ultrasound. Multiplanar reformatting of 3D-UBS volume allows visualization of oblique views, (green-**c** and red-**d**), that provide more accurate size and location information.

Experience with the 3D-UBS helped us identify some unique advantages as compared to conventional 2D handheld ultrasound. With 3D-UBS, the operator simply placed the probe on top of the eye, selected the start and end point for the scan via the control software, and images were automatically acquired by the Clarius imaging software. As compared to conventional 2D hand-held ultrasound, the 3D-UBS has unique advantages. (1) Results are not dependent upon the skill of the operator. The operator does not even need to know eye anatomy. (2) The 3D acquisition is done in a prescribed, gentle way, increasing safety. Potential slippage of a hand-held device is not present. (3) As a full 3D acquisition is obtained, one can apply multiplanar reformatting to optimally visualize an ultrasound plane. (4) A unique en face view is possible which greatly aids the determination of ophthalmological structures^19,20^. (5) Volumetric measurements are possible. (6) With 3D, it is easy to determine the location of an IOFB relative to other critical structures (e.g., the retina, lens, etc.) for planning interventions. Figure 4 shows a comparison of visualization between 2D ultrasound and 3D-UBS. 2D ultrasound provides a limited view of the ocular anatomy, as shown in Fig. 4a. With 3D-UBS, en face view of the eye can be visualized which is not possible with 2D. A sample en face view (Fig. 4b) shows the location of IOFB with respect to intraocular lens. Multiplanar reformatting allows optimal view of the IOFB (Fig. 3c and d), and the physical dimension as well as location of the IOFB can be calculated. We made measurements in the 3D-UBS volume by using measurement tools available in Amira software package (Thermo Fisher Scientific, Waltham, MA). Figure 4c shows that the minimum distance between the lens and IOFB as 4.06 mm. The volume of the IOFB was measured at 14.14 mm^3^ via manual annotation. The shape of the wooden IOFB was measured with a caliper and was assumed to be a frustum of a cone with top base radius $r$ = 0.25 mm, larger base radius $R=$ 1.05 mm, and height $h=$ 9.96 mm. The analytical volume was calculated as 14.89 mm^3^ using the formula, $V_{frustum} = \frac{1}{3}\pi h\left( {R^{2} + r^{2} + Rr} \right)$. The difference between manual and analytical volume was ~ 5%.

### Visualization and detectability of different IOFBs using 3D-UBS

We created digital phantoms of IOFB immersed in the vitreous humor (details in Section “Digital phantom preparation”). From these digital phantoms and ex-vivo models, we calculated the SNR_Rose_ and CNR between the 3D-UBS and CT systems.

#### Wooden IOFB in 3D-UBS and CT

**Figure 5:**
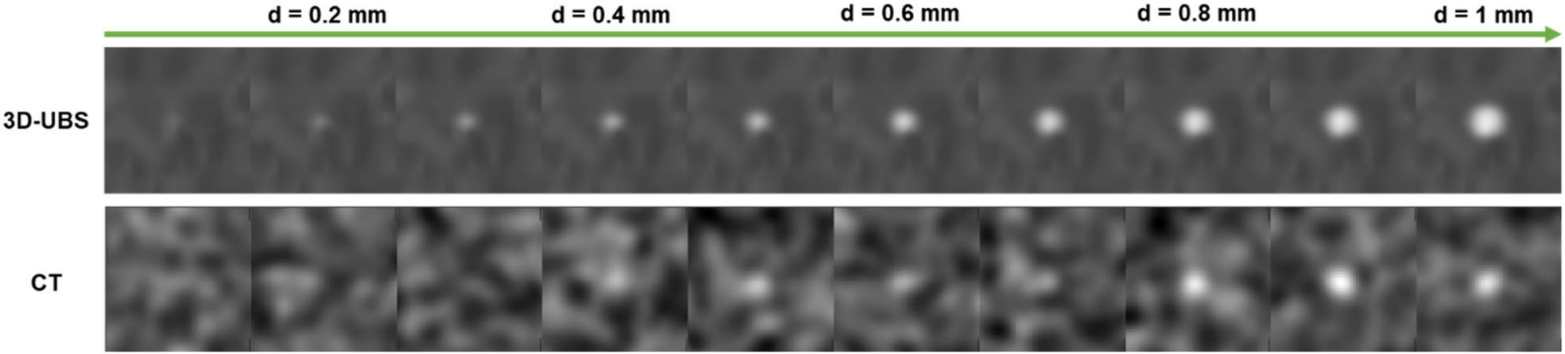
Visualization of wooden IOFBs in 3D-UBS and CT using digital phantom. Wooden IOFBs of increasing diameters (0.1–1 mm) are embedded in vitreous humor. 3D-UBS is superior in small wood IOFB visualization.

Digital phantom images (${I}_{dp-wood}$) show that 3D-UBS has better qualitative visualization and quantitative detectability for wooden IOFBs. . Figure 5 shows a visual comparison in small IOFB visualization between 3D-UBS and CT images of the wooden digital phantom experiment. A high acoustic impedance mismatch between vitreous-wood leads to higher ultrasound reflection. Wood due to being porous, has a similar x-ray attenuation coefficient (HU) as vitreous humor. In our experiments, the mean ± SD of HU values for wood was 40 ± 43. 3D-UBS has a higher resolution compared to clinical CT, in terms of PSF. The blurring effect of PSF_CT_ impacted visualization of small wooden IOFBs more adversely, compared to PSF_3D-UBS_. Although larger wooden IOFBs (> ~ 600 µm), were visible in CT, 3D-UBS provided better visualization.

**Figure 6:**
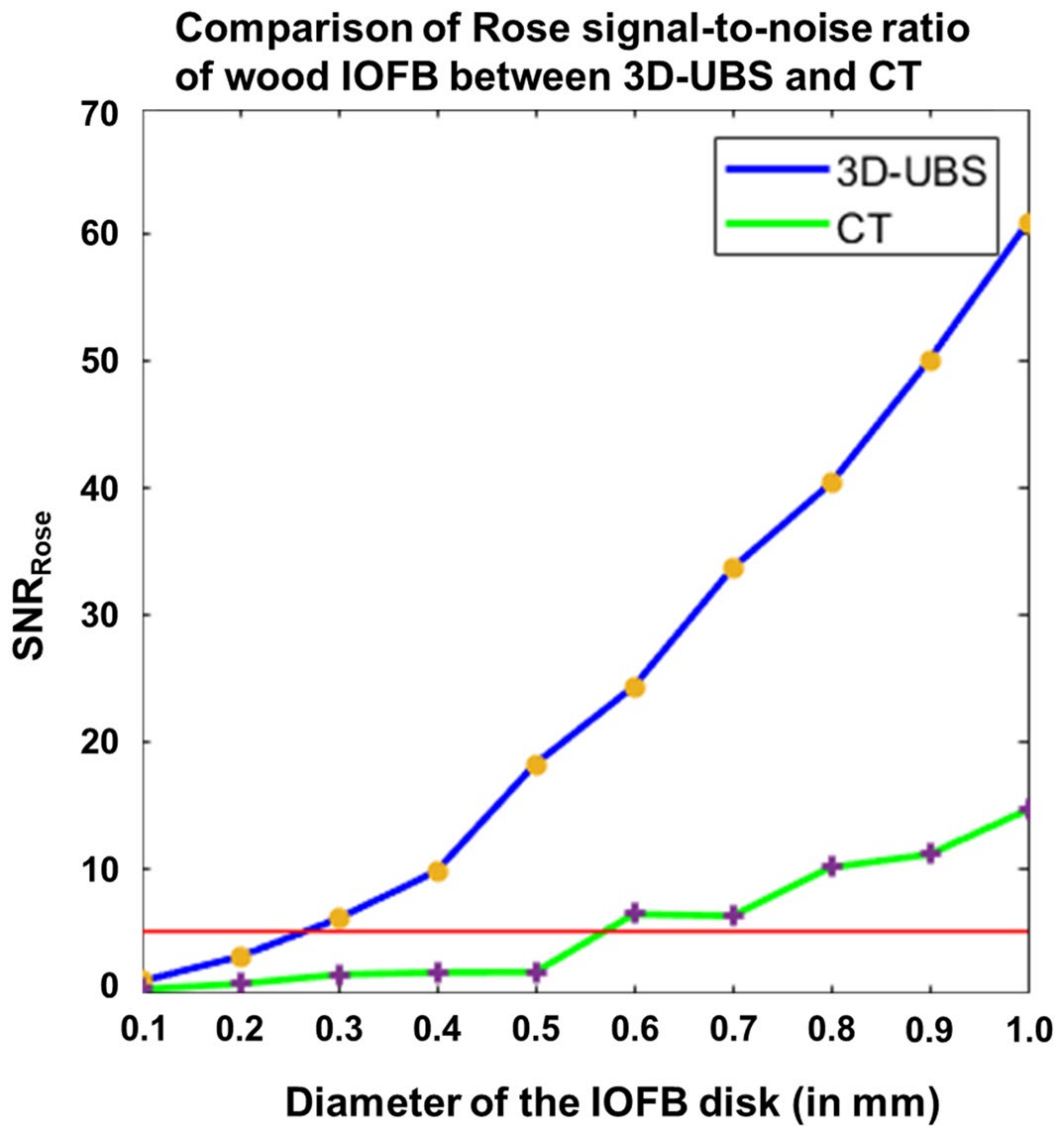
Comparison of Rose signal-to-noise ratio (SNR_Rose_) between 3D-UBS and CT images of wooden IOFB digital phantoms. Digital phantoms consist of a circular disk of increasing diameter in vitreous humor. The red line indicates the Rose criterion for object detectability (SNR-rose = 5). 3D-UBS is superior at every diameter in wooden object detectability compared to CT and detects objects smaller than ~ 600 µm. CT can detect wooden IOFBs > 600 µm in diameter.

Digital phantom images also (${I}_{dp-wood}$) show that for detecting wooden IOFBs in vitreous, 3D-UBS is superior compared to CT. Quantitatively, object detectability is measured using SNR_Rose_, with SNR_Rose_ > 5 indicating positive detection^10^. Figure 6 shows the quantitative comparison between 3D-UBS and CT, in detecting wooden IOFBs of increasing size (< 1 mm). 3D-UBS provides higher detectability compared to CT at every size and can also detect objects above than ~ 250 µm in diameter. CT can only detect wooden IOFBs above ~ 600 µm in diameter. When the wooden IOFB diameter increases, the difference in SNR_Rose_ between 3D-UBS and CT decreases, but 3D-UBS provides better detectability.

**Figure 7:**
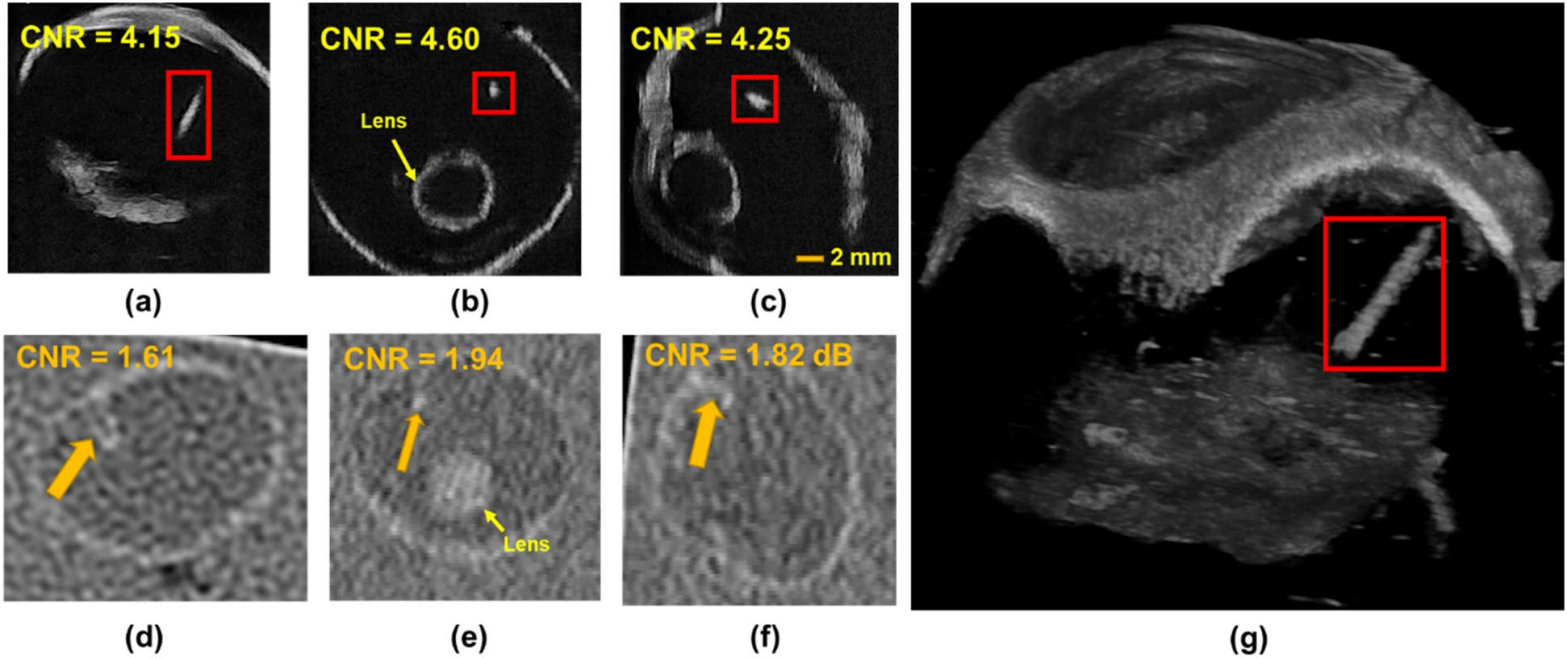
Visualization of wooden IOFB in 3D-UBS and CT. (**a**)–(**c**) and (**d**)–(**f**) show 2D views of the same wooden IOFB using 3D-UBS (red box) and CT (yellow arrow), respectively. Due to vitreous being anechoic to ultrasound, 3D-UBS shows superior contrast compared to CT in all three planes. (**g**) shows 3D visualization of the entire wooden IOFB.

Ex-vivo imaging (${I}_{exvivo-wood}$) showed that 3D-UBS is better at wooden IOFB visualization compared to CT. Figure 7a–c and d–f show 2D views of the same wooden IOFB using 3D-UBS and CT, respectively. Wooden IOFB was seen in all three planes of 3D-UBS, however, it was not always possible to locate the object in all three planes of CT, depending on the HU values and size of the object. 3D-UBS consistently showed higher CNR compared to CT in all three planes. In each of these planes, 3D-UBS showed 2.3–2.6 times more contrast compared to CT. Our theoretical digital phantom achieved an SNR_Rose_ of 151.2 in 3D-UBS for a wooden IOFB of 2 mm diameter. Using Eqs. (3) and (4), we calculated the CNR as 4.17, which is < 4%, compared to the experimental mean CNR of 4.33, implying good modeling of acoustic response. Figure 7g shows that 3D-UBS also provided whole IOFB visualization which can be used to locate IOFB with respect to ocular structures of interest.

#### Plastic IOFB in 3D-UBS and CT

**Figure 8:**
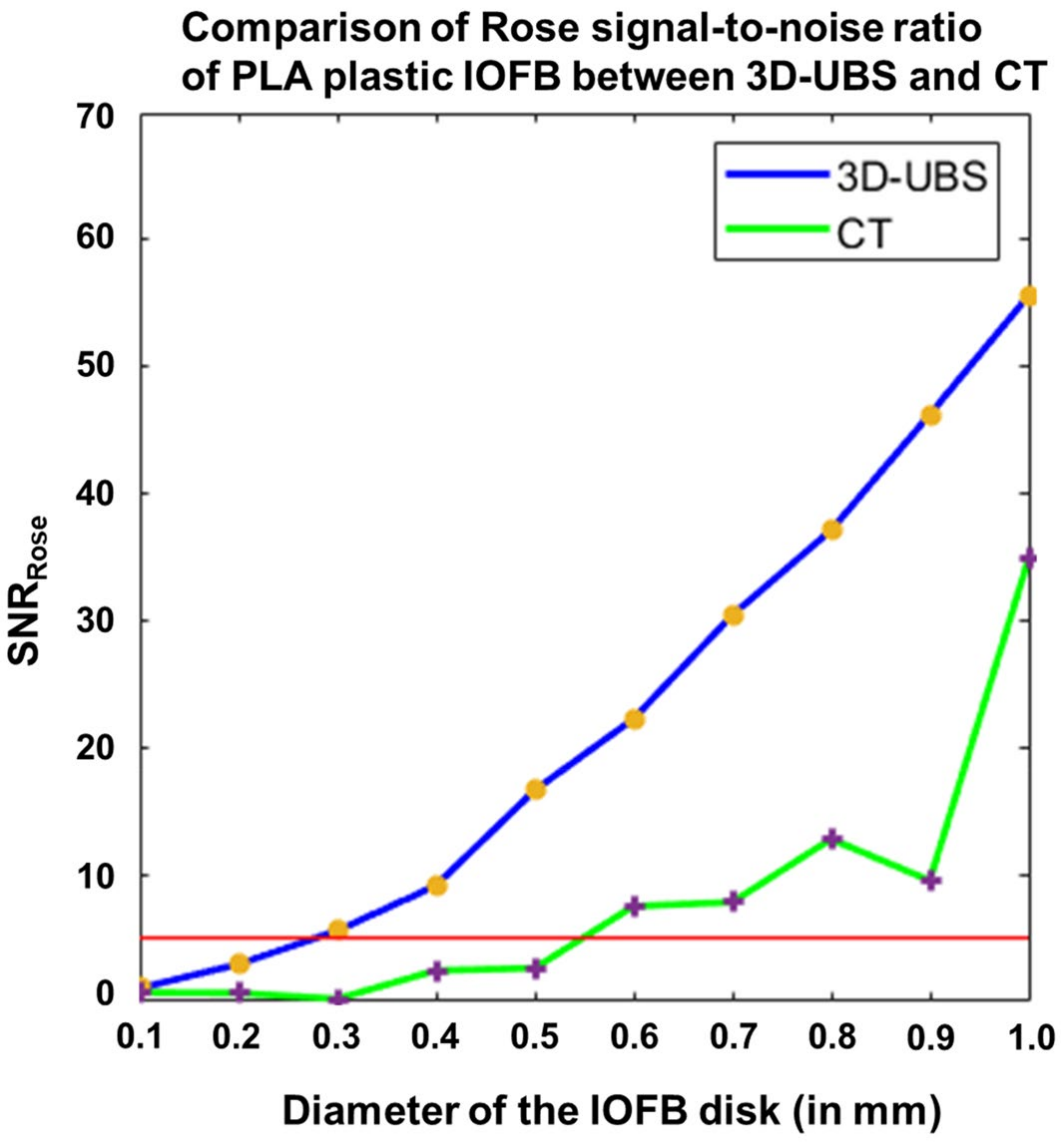
Comparison of Rose signal-to-noise ratio (SNR_Rose_) between 3D-UBS and CT in images of plastic IOFB digital phantom. 3D-UBS shows consistently higher detectability in detecting plastic IOFBs compared to CT and can detect objects within a diameter of 300–550 µm. CT can detect plastic IOFBs > 550 µm in diameter.

Digital phantom images (${I}_{dp-plastic}$) show that 3D-UBS has better detectability of plastic IOFBs at all sizes and especially can detect smaller plastic IOFBs (300–550 µm) with a better contrast than CT. Figure 8 shows a quantitative comparison of detectability in terms of SNR_Rose_ between 3D-UBS and CT in plastic IOFB digital phantom. This occurs because of the high acoustic impedance mismatch between vitreous-IOFB surfaces leading to higher ultrasound reflection^21–23^. However, plastic (PLA) has a similar x-ray attenuation coefficient (HU) as vitreous humor. In our experiments, the mean ± standard deviation (SD) of HU values for PLA plastic were − 20 ± 27. CT did not result in an SNR_Rose_ > 5 for a plastic IOFB unless the object diameter is sufficiently large (> 500 µm). With larger objects, the difference in SNR_Rose_ decreased, however, 3D-UBS showed consistently higher SNR or detectability.

**Figure 9:**
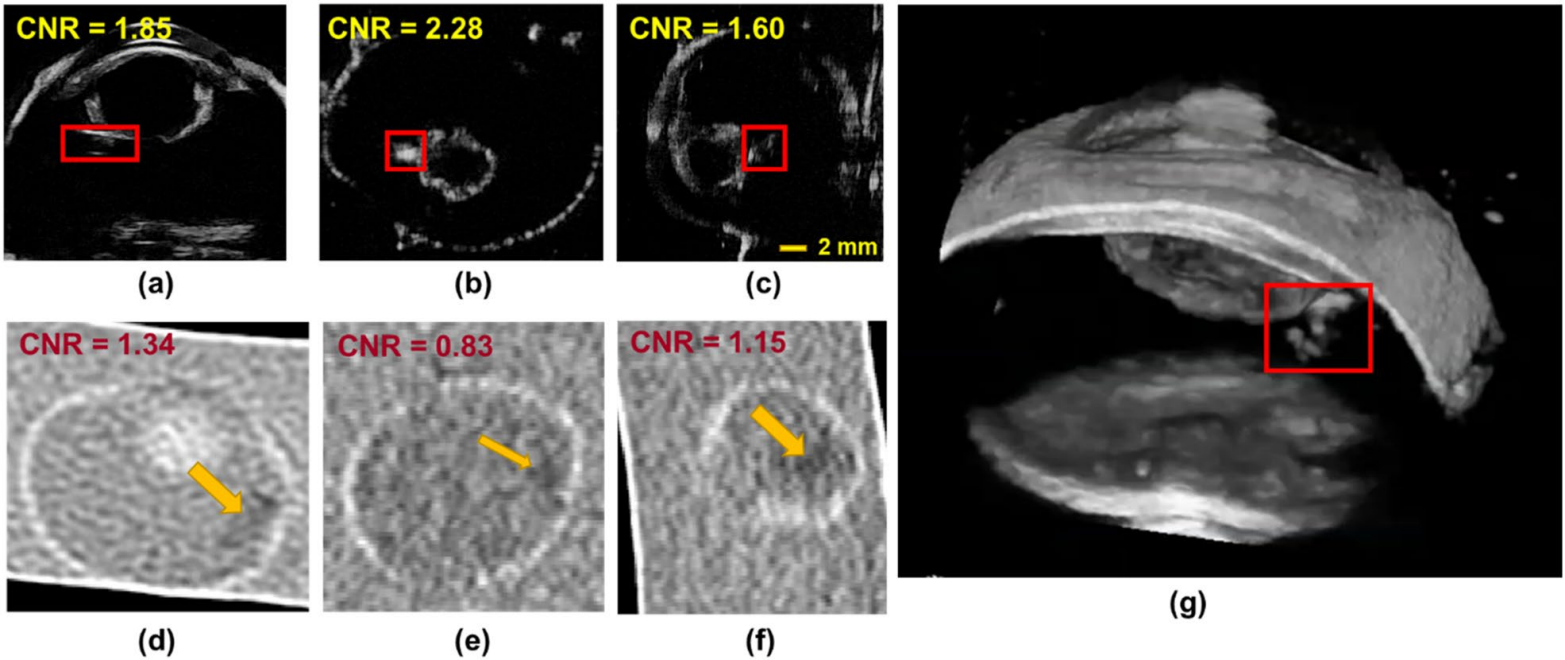
Visualization of plastic IOFB in 3D-UBS and CT. (**a**)–(**c**) and (**d**)–(**f**) show 2D views of the same plastic IOFB using 3D-UBS (red box) and CT (yellow arrow), respectively. Due to the vitreous being anechoic to ultrasound, 3D-UBS shows superior contrast compared to CT in all three planes. Out-of-plane contrast of the plastic IOFB using CT is very poor compared to 3D-UBS, making it difficult to identify the IOFB properly. En face views (b) vs (**e**) shows the highest (2.75×) contrast-to-noise ratio (CNR) improvement. (**g**) shows 3D visualization of the entire plastic IOFB. Plastic IOFB causes acoustic shadowing due to high impedance mismatch, occluding some parts of the IOFB as seen in (**g**).

Ex-vivo imaging (${I}_{exvivo-plastic}$) showed that 3D-UBS is better at plastic IOFB visualization compared to CT. Figure 9a–c and d–f show 2D views of the same plastic (PLA) IOFB using 3D-UBS and CT, respectively. In each of these planes, 3D-UBS showed superior contrast compared to CT. We observed lower in-plane contrast (CNR = 1.38) in detecting plastic IOFB using CT. Out-of-plane contrast of the plastic IOFB using CT was very poor compared to 3D-UBS, making it difficult to identify the IOFB properly. En face views (Fig. 9b and e) showed the highest contrast-to-noise ratio (CNR) improvement (2.75× improvement). Figure 9g shows 3D visualization of the entire plastic IOFB. Plastic IOFB causes acoustic shadowing due to high impedance mismatch, occluding some parts of the IOFB as seen in Fig. 9g. For a plastic object of 3 mm diameter, we calculated CNR = 3.97 (from SNR_Rose_ using Eqs. 3 and 4), which is higher than experimental CNR we observed (mean CNR = 1.91). This is also likely due to the acoustic shadowing that decreases mean grayscale signal values within region-of-interest, ${\mu }_{obj}$ resulting in lower CNR.

#### Metal and glass IOFB in 3D-UBS and CT

**Figure 10:**
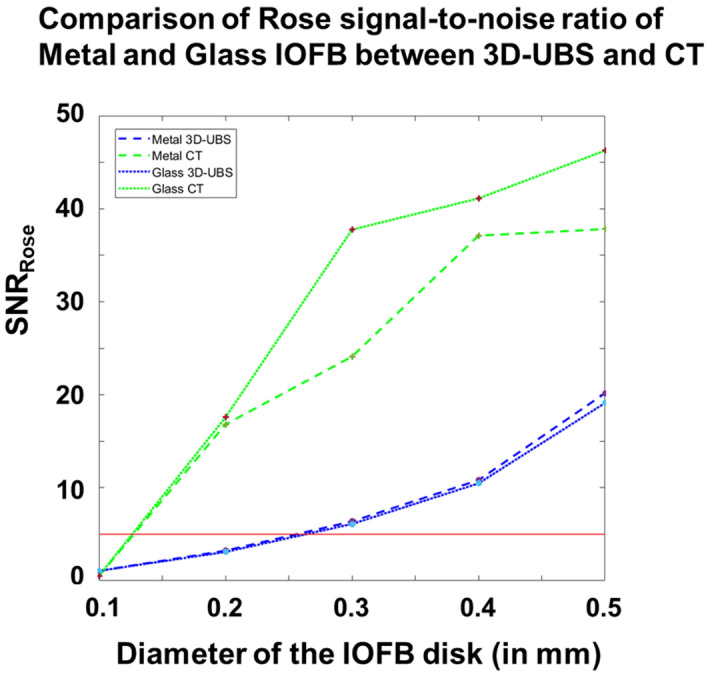
Comparison of Rose signal-to-noise ratio (SNR_Rose_) between 3D-UBS and CT in images of glass and metal IOFB digital phantom. Due to high HU values of glass and metal, the contrast of the CT even when the object is smaller (diameter ~ 150 µm) is high, resulting in a high SNR_Rose_ compared to 3D-UBS. However, 3D-UBS maintains high detectability, as the specific acoustic impedances of those materials are also high, compared to vitreous humor.

Figure 10 shows that materials with high HU and high specific acoustic impedance, compared to vitreous, i.e., metal or glass are highly detectable in both CT and 3D-UBS, evidenced by SNR_Rose_ > 5. CT has an advantage over 3D-UBS in detecting small metal or glass IOFBs (diameter within 150–50 µm). 3D-UBS shows detectability above 250 µm. High HU of such materials causes mean signal within region-of-interest, ${\mu }_{obj}$ to increase relative to the background, leading to higher contrast. 3D-UBS is also capable of detecting small metal and glass IOFBs (diameter > 250 µm) with SNR_Rose_ > 5.

**Figure 11:**
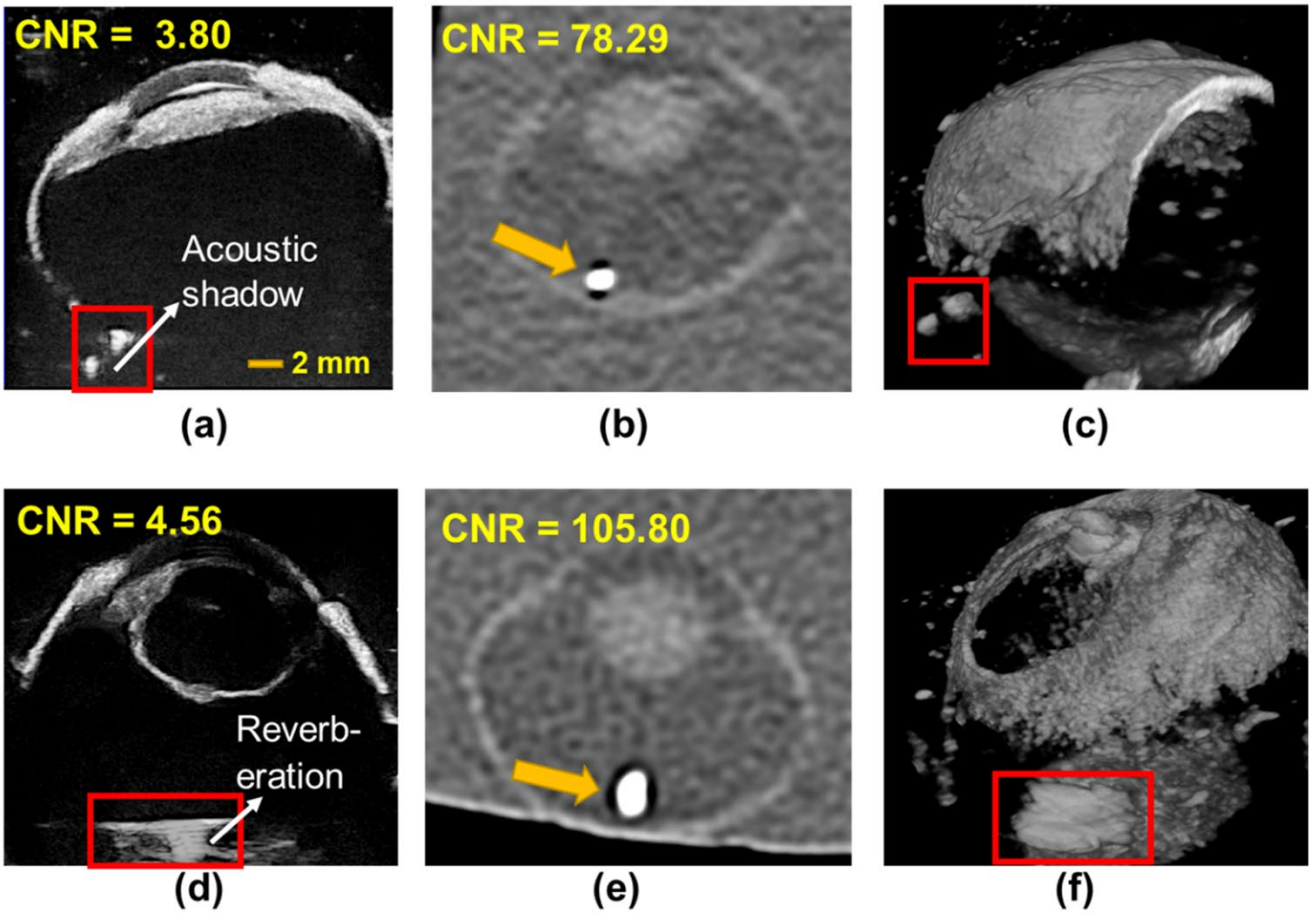
Visualization of metal and glass IOFB in 3D-UBS and CT. (**a**)–(**b**) and (**d**)–(**e**) show 2D views of the same metal and glass IOFB using 3D-UBS (window/level = 255/127) and CT (window/level = 450/75), respectively. (**c**) and (**d**) show 3D volume rendering of the IOFBs in 3D-UBS.

In ex vivo imaging, 3D-UBS and CT both identified metal and glass with high contrast. Figure 11 shows that the contrasts of both materials with CT were high, as their linear X-ray attenuation coefficients are high. The presence of highly attenuating materials i.e., glass, metal cause CT artifacts (i.e., photon starvation), resulting in a dark region around the IOFBs. This coupled with high HU values of the materials provides high CNR for metal or glass IOFBs. However, the effect in terms of IOFB detection was minimal, as 3D-UBS also provides good contrast. We observed acoustic shadowing for metal, and reverberation artifacts for glass IOFBs in 3D-UBS. Although the presence of such artifacts can help detect IOFBs, they prevent accurate measurements of the size of the foreign body.

## Discussion and conclusion

We developed a novel 3D ultrasound B-scan (3D-UBS) imaging system to particularly address diagnostic challenges when the eye has been perforated with foreign bodies. 3D-UBS demonstrates higher SNR_Rose_, both experimentally and analytically, as compared to CT, only for wood and plastic IOFBs. This makes it much easier to visualize wood and plastic IOFBs with 3D-UBS than that with CT. 3D visualizations in 3D-UBS are typically very striking and unambiguous. Wood and plastic IOFBs were visible in all three planes with 3D-UBS, but not with CT. 3D-UBS also demonstrated higher detectability when IOFBs were smaller than 600 and 500 µm in diameter, for wood and plastic respectively. 3D-UBS is better at differentiating smaller IOFBs compared to clinical CT due to its narrower PSF, hence its higher resolution. In the case of multiple smaller IOFBs, 3D-UBS should be able to differentiate those as separate objects due to its higher resolution. Wider PSF leads to blurring, therefore a narrower PSF is preferred. Blurring reduces contrast, which reduces CNR which reduces detectability (i.e., SNR_Rose_). In glass and metal, we see higher SNR_Rose_ and CNR in CT, compared to 3D-UBS. The numerical advantage is due to high HU values of highly attenuating materials such as metal or glass compared to vitreous, leading to higher contrast. Contrast is also bit-depth dependent. Clinical CT images have a bit depth of 12-bits, therefore can accommodate a maximum contrast, $(|{\mu }_{obj}-{\mu }_{bg}|$) of 4095. Clinical B-scan ultrasound images have a bit depth of 8-bits leading to a maximum possible contrast of 255. Additionally, there are caveats to the application of SNR_Rose_. SNR_Rose_ can be derived from signal detection theory assuming white noise^16^. Both ultrasound and CT have correlated noise, so Rose is approximately applied. Regardless, the major conclusions from this analysis are valid. We have previously demonstrated clinical 3D ultrasound in anterior segment imaging^19^. In this study, we evaluated our 3D-UBS system for ex vivo porcine eyes only. 3D-UBS can be extended to human subjects in future using the same safety, although that is outside the scope of our current study.

Our proposed system aims to enable easy, non-expert utilization for identifying ocular injuries at the point of care. Through the integration of ultra-high frequency array technologies and advanced processing, we created easily interpretable renderings of the entire globe with injuries. These combined advancements hold the promise of streamlining triage and providing a timely diagnosis of vision-threatening ocular injuries and neural conditions, with post-diagnosis potential for surgical planning and treatment response assessment.

## Data Availability

The datasets generated during and/or analyzed during the current study are available from the corresponding author on reasonable request.
